# A Phase 1 study of gefitinib combined with durvalumab in EGFR TKI-naive patients with *EGFR* mutation-positive locally advanced/metastatic non-small-cell lung cancer

**DOI:** 10.1038/s41416-020-01099-7

**Published:** 2020-10-05

**Authors:** Benjamin C. Creelan, Tammie C. Yeh, Sang-We Kim, Naoyuki Nogami, Dong-Wan Kim, Laura Q. M. Chow, Shintaro Kanda, Rosemary Taylor, Weifeng Tang, Mei Tang, Helen K. Angell, Martine P. Roudier, Marcelo Marotti, Don L. Gibbons

**Affiliations:** 1 Department of Thoracic Oncology, H. Lee Moffitt Cancer Center and Research Institute, 12902 Magnolia Drive, FOB-1, Tampa, FL 33612 USA; 2 Translational Medicine, Oncology R&D, AstraZeneca, 35 Gatehouse Drive, Waltham, MA 02451 USA; 3 Department of Oncology, Asan Medical Center, University of Ulsan College of Medicine, 88, Olympic-ro 43-gil, Songpa-gu, Seoul, 05505 South Korea; 4 Department of Thoracic Oncology, National Hospital Organization Shikoku Cancer Center, 160 Minami-Umemoto-cho, Matsuyama City, 791-0280 Japan; 5 Department of Internal Medicine, Seoul National University Hospital, 101 Daehak-ro, Jongno-gu, Seoul, 03080 Republic of Korea; 6 Department of Medicine, Division of Oncology, University of Washington/Seattle Cancer Care Alliance, Seattle, WA 98109 USA; 7 Department of Comprehensive Cancer Therapy, Shinshu University School of Medicine, 3-1-1 Asahi Matsumoto, Nagano, 390-8621 Japan; 8 Oncology, AstraZeneca, Academy House, 132-136 Hills Road, Cambridge, CB2 8PA UK; 9 Clinical Pharmacology and Safety Assessment, AstraZeneca, One Medimmune Way, 101 ORD, 2001D, Gaithersburg, MD 20878 USA; 10 Translational Medicine, Oncology R&D, AstraZeneca, Darwin Building, Unit 310, Cambridge Science Park, Milton Road, Cambridge, CB4 0WG UK; 11 Departments of Thoracic/Head and Neck Medical Oncology and Molecular and Cellular Oncology, The University of Texas MD Anderson Cancer Center, 1515 Holcombe Boulevard, Houston, TX 77030 USA

**Keywords:** Tumour biomarkers, Cancer immunotherapy, Non-small-cell lung cancer, Targeted therapies

## Abstract

**Background:**

EGFR tyrosine kinase inhibitors (TKIs) induce cytolysis and release of tumour proteins, which can stimulate antigen-specific T cells. The safety and efficacy of durvalumab and gefitinib in combination for TKI-naive patients with advanced *EGFR*m NSCLC was evaluated.

**Methods:**

This Phase 1 open-label, multicentre trial (NCT02088112) was conducted in 56 patients with NSCLC. Dose expansion permitted TKI-naive patients, primarily with activating L858R or Ex19del *EGFR*m. Arms 1 + 1a received concurrent therapy; Arm 2 received 4 weeks of gefitinib induction followed by concurrent therapy.

**Results:**

From dose escalation, the recommended dose of durvalumab was 10 mg/kg Q2W with 250 mg QD gefitinib. Pharmacokinetics were as expected, consistent with inhibition of soluble PD-L1 and no treatment-emergent immunogenicity. In dose expansion, 35% of patients had elevated liver enzymes leading to drug discontinuation. In Arms 1 + 1a, objective response rate was 63.3% (95% CI: 43.9–80.1), median progression-free survival (PFS) was 10.1 months (95% CI: 5.5–15.2) and median response duration was 9.2 months (95% CI: 3.7–14.0).

**Conclusions:**

Durvalumab and gefitinib in combination had higher toxicity than either agent alone. No significant increase in PFS was detected compared with historical controls. Therefore, concurrent PD-L1 inhibitors with gefitinib should be generally avoided in TKI-naive patients with *EGFR*m NSCLC.

## Background

Epidermal growth factor receptor (EGFR) tyrosine kinase inhibitors (TKIs) are the preferred first-line therapy for patients with metastatic non-small cell lung cancer (NSCLC) harbouring sensitising *EGFR* mutations.^1^ Advances in the chemistry of EGFR TKIs offer the potential for improved response and survival.^1–4^ However, acquired resistance to EGFR TKIs remains largely inevitable, with disease progression typically occurring within 2 years.^1,5–7^ Furthermore, programmed cell death-1 (PD-1) axis blockade as monotherapy has attenuated efficacy in *EGFR* mutation-positive lung cancer, with reported response rates of ≤10%.^8–12^ Therefore, novel strategies to boost the durability of TKI responses are urgently needed. Targeting EGFR may promote an inflamed tumour microenvironment through engagement of Fc-γ receptors on immune cells, thereby boosting T cell cross-priming and antigen presentation.^13^ EGFR TKIs cause immunogenic apoptosis of tumour cells,^14^ releasing aberrant intracellular antigens and recruiting T cells via interferon-γ-induced major histocompatibility complex (MHC) class I presentation.^15^ This phenomenon further promotes expression of T cell chemoattractants, chemokine (C-C motif) ligand 2 (CCL2), CCL5 and chemokine (C-X-C motif) ligand 10.^16^ Gefitinib treatment has been shown to boost CD8^+^ T cell recruitment via MHC I upregulation and antigen cross-presentation within the tumour.^17–21^ Interestingly, programmed cell death ligand-1 (PD-L1)-expressing clones have been identified as EGFR TKI-resistant tumours.^22,23^ In fact, PD-L1 expression may predict poor response and lower survival rates with EGFR TKI monotherapies for patients with activating *EGFR* mutations.^24–28^ Therefore, PD-L1 immune checkpoint inhibition may be an attractive combination to partner with gefitinib in the first-line setting.

Durvalumab is a selective, high-affinity human IgG1κ monoclonal antibody that blocks PD-L1 binding to PD-1 and CD80.^29^ Objective response rates of approximately 12% have been reported with durvalumab monotherapy in EGFR TKI-resistant tumours with strong PD-L1 expression.^30^ We hypothesised that the combination of gefitinib with durvalumab would exert therapeutic synergy by inducing differentiation and engraftment of memory T cells immediately after initial TKI treatment, therefore inducing more durable clinical remissions with the EGFR TKI. We performed a Phase 1 study to assess the safety and efficacy of concurrent gefitinib and durvalumab for the treatment of TKI-naive patients with *EGFR* mutation-positive NSCLC.

## Methods

### Study design

**Fig. 1:**
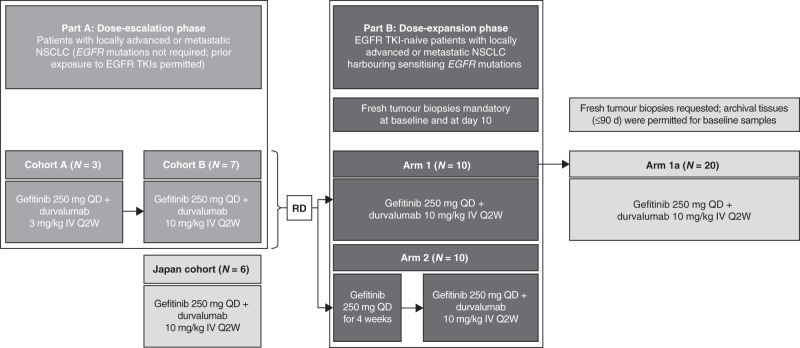
Study design. d days, EGFR epidermal growth factor receptor, IV intravenous, *N* number of patients assigned to treatment, NSCLC non-small cell lung cancer, QD once daily, Q2W once every 2 weeks, TKI tyrosine kinase inhibitor.

This was an open-label, multicentre Phase 1 trial (NCT02088112) with a modified 3 + 3 dose-escalation phase followed by a multi-arm dose-expansion phase, conducted at seven sites in the US, Japan and Korea. A fixed dose of gefitinib 250 mg daily (QD) was selected for all cohorts, based upon the established maximal biologic activity in vivo.^31^ In the dose-escalation phase (Fig. 1), patients received gefitinib 250 mg QD plus durvalumab (MEDI4736) at 3 or 10 mg/kg intravenous (IV) every 2 weeks (Q2W). Cohort A received durvalumab at 3 mg/kg IV Q2W. Next, a subsequent Cohort B and a Japan Cohort received durvalumab at 10 mg/kg. Dose-limiting toxicity (DLT) was defined as any possible treatment-related Grade ≥3 adverse event (AE), regardless of duration, within the first treatment cycle of 28 days. This included any Grade 4 immune-mediated AEs that were not attributable to lung cancer.

The dose-expansion phase comprised three arms. Patients enrolled in Arm 1 received gefitinib 250 mg QD plus durvalumab 10 mg/kg IV Q2W. Arm 1 was intended to address whether concurrent gefitinib and durvalumab could achieve a more durable response than historical gefitinib monotherapy. Patients enrolled in Arm 2 received gefitinib monotherapy induction for 28 days followed by concurrent gefitinib plus durvalumab. The rationale for the induction Arm 2 was that gefitinib would induce tumour autophagy with MHC class I cross-presentation of tumour antigens and the activation of CD8+ T cells over time, thereby priming T cells for durvalumab at Day 28.^32^ Arm 1a was later added to the study protocol to further explore the safety and clinical activity of the dosing schedule used in Arm 1. For all cohorts, concurrent therapy was given for up to 12 months; and thereafter patients continued with gefitinib monotherapy until disease progression.

### Patients

Screening was conducted between March 2014 and February 2015. Patients were required to have tissue-confirmed metastatic or advanced NSCLC by AJCC seventh edition cancer staging criteria^33^ that was not amenable to definitive surgery or radiation. The dose-escalation phase permitted patients with any relapsed/refractory NSCLC or those who were intolerant or not eligible for any line of standard treatment. This cohort did not require an activating *EGFR* mutation, and prior treatment with EGFR TKIs was permitted. The dose-expansion phase permitted only EGFR TKI-naive patients with tumours harbouring a sensitising *EGFR* mutation. Mandatory tumour biopsies were required at screening and on Day 10 of treatment. Patients in Arm 1a were permitted to submit an archival tissue sample in place of the screening sample, if collected within 90 days prior to the first dose (*N* = 12). For additional patient eligibility criteria, please see Supplementary Data 1. All patients provided written informed consent; the final protocol was approved by the local ethics committee or Institutional Review Board at each site.

### Assessments

In the dose-escalation phase, the primary objective was to assess the safety and tolerability of concurrent gefitinib plus durvalumab and establish a recommended dose of durvalumab for use in the dose-expansion phase. In the dose-expansion phase, the primary objective was to confirm the safety and tolerability of the gefitinib plus durvalumab combination in the intent-to-treat population, for use in future studies. Secondary objectives included pharmacokinetics, durvalumab immunogenicity, durvalumab pharmacodynamics and efficacy.^34^ Efficacy endpoints included overall response rate (ORR), disease control rate (DCR), DCR at 16 weeks, duration of response and progression-free survival (PFS). Overall survival was added as a protocol amendment later in the course of the study; however, few patients could subsequently consent to this protocol amendment.

*EGFR* mutation was determined by local site laboratories. Exploratory objectives included correlation of baseline tumour PD-L1 expression with efficacy. Tumour cell (TC) PD-L1 immunohistochemistry (Ventana, clone SP263^35^) was blindly scored by a pathologist using an established scoring protocol.^36^ An exploratory cut-point (PD-L1 TC ≥20%) was empirically chosen as it provided more meaningful group numbers for analysis (PD-L1 TC ≥20%: *N* = 12; PD-L1 TC <20%: *N* = 24) than the more typical cut-off of PD-L1 TC ≥25% (*N* = 7; PD-L1 TC: <25%: *N* = 29). Safety and tolerability were assessed in the safety population of all patients who received at least one dose of study medication. Pharmacokinetics were assessed in all patients who had at least one measurable post-dose pharmacokinetic concentration. Tumour response was assessed in all patients with a baseline tumour assessment who received study medication. Study sample size was based on the desire to obtain adequate tolerability, safety, pharmacokinetic and pharmacodynamic data while exposing as few subjects as possible to the investigational product and procedures. Further details are provided in Supplementary Data 1.

## Results

### Patient characteristics

Of the 70 patients screened, 56 were eligible and treated (Supplementary Fig. 1). Patient demographics and baseline characteristics are reported in Supplementary Table 1. Overall, in the dose-escalation and dose-expansion phases, patients had a median age of 61 years (range: 27–83) and 55% were female. There was a slightly higher prevalence of Asian (55%) patients, compared with Caucasian (43%) and Black (4%) patients. *EGFR* mutation status in the dose-expansion phase included 2 patients with exon 18 mutations, 21 with exon 19 deletions, 16 with exon 21 L858R and 1 with L858R/L861Q.

### Dose-escalation phase

**Fig. 2:**
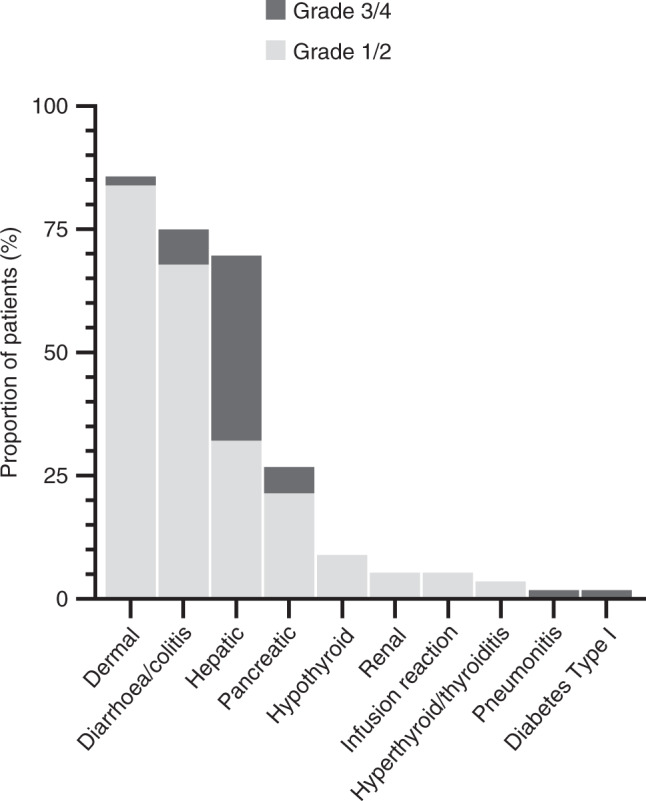
Adverse events of special interest by CTCAE grade (safety population, dose escalation and expansion). Maximum CTCAE grade is shown for all cohorts, including Cohort A (*N* = 3), Cohort B (*N* = 7), Japan Cohort (*N* = 6), Arm 1 (*N* = 10), Arm 1a (*N* = 20) and Arm 2 (*N* = 10). CTCAE Common Terminology Criteria for Adverse Events, *N* number of patients assigned to treatment.

In the dose-escalation phase, three patients in Cohort A received durvalumab 3 mg/kg IV Q2W and 13 patients in Cohort B and a Japan Cohort received durvalumab 10 mg/kg IV Q2W. No DLTs were reported, and the maximum tolerated dose of durvalumab was not reached. The recommended dose for the dose-expansion phase was thus deemed to be 10 mg/kg IV Q2W. For the subset of patients in the dose-escalation phase with *EGFR* mutations (11/16), the majority were either *EGFR*^L858R^ (5/9) or *EGFR*^ΔEx19^ (4/9) (Supplementary Table 1); some had prior TKI treatment and others did not. AEs are summarised in Supplementary Table 2; common AEs included diarrhoea (69%), transaminitis (44%), fatigue (44%) and nausea (44%). AEs of special interest are shown in Fig. 2. In the dose-escalation phase, no diarrhoea above Grade 2 was reported; one patient reported Grade 3 dermatitis/rash. However, after completion of the 28-day DLT evaluation period, a further 8 hepatic events were reported, including 3 Grade 3/4.

Overall, serious AEs were reported in 50.0% of patients and Grade 3/4 AEs were reported in 68.8% of patients. Five patient deaths occurred in the dose-escalation phase. The primary causes of death were NSCLC (*N* = 3), pericardial effusion (*N* = 1) and suicide (*N* = 1) and were not considered to be treatment related by the investigator. Most patients discontinued combination treatment after the DLT evaluation period (94%); AEs were the most common cause for discontinuation. Eight patients (50%) discontinued treatment as a result of AEs, with elevated alanine aminotransferase (ALT) being the most frequently reported AE leading to discontinuation (*N* = 3). In the dose-escalation phase, response data were not considered meaningful due to the wide heterogeneity of these patients in terms of disease stage, *EGFR* mutation status and absence/presence of prior TKI therapy.

### Safety and tolerability in the dose-expansion phase

In the dose-expansion phase, all patients (*N* = 40) were TKI naive and had a sensitising *EGFR* mutation (Supplementary Table 1). Ten patients were each enrolled into Arm 1 and Arm 2. As no patients in Arm 1 had AEs leading to treatment discontinuation, a further 20 patients were enrolled into the concomitant dosing group, Arm 1a (Supplementary Table 2). Common AEs included diarrhoea (78%), elevated ALT (60%) and aspartate aminotransferase (AST) (45%), rash (53%) and pruritus (45%). The most common causally related AEs are summarised in Supplementary Table 3. No deaths were treatment related. Common AEs of special interest were dermatitis/rash, diarrhoea/colitis and hepatic events; 17 patients had high-grade hepatic events (Fig. 2). Management of elevations in ALT and AST primarily consisted of dose interruptions and prompt serial rechecking of AST/ALT levels. If AST/ALT levels continued to increase, despite cessation of both drugs, then administration of a corticosteroid, usually 1 mg/kg oral prednisone, was initiated. Once AST/ALT levels improved, corticosteroid dose was tapered over the course of 3–5 weeks. With this management, hepatic AEs of special interest resolved for most patients (87.1%) but remained a pervasive problem during the trial. The majority of patients discontinued gefitinib plus durvalumab combination treatment before the full 1-year treatment period ended (*N* = 28; 70.0%). Of these, 17 patients discontinued as a result of AEs, most frequently due to elevated ALT (47% [8/17]) and AST (35% [6/17]). Although these patients stopped combination treatment, they continued on gefitinib monotherapy. Patients with hepatic AEs that led to treatment discontinuation are shown in Table 1.

**Table 1:** Patients with hepatic adverse events leading to treatment discontinuation.

Patient (cohort)	AE (max. CTCAE grade)	Onset	ALT	AST	BIL	Last available value	TRAE^a^	Outcome
1 (Arm 1a)	DILI^b^ (Grade 4)	D41	D41: 86 U/L D55: 1639 U/L	D41: 76 U/L D55: 1208 U/L	D41: 27 µmol/L D55: 69 µmol/L	**FU (D125):** ALT: 10 U/L; AST: 15 U/L; BIL: 21 µmol/L	No	Recovered/resolved
2 (Arm 1a)	Transaminases increased^b^ (Grade 4)	D44	D44: 992 U/L	D44: 829 U/L	D44: 24 µmol/L	**FU (D86):** ALT: 13 U/L; AST: 22 U/L	Yes	Recovered/resolved
3 (Arm 1a)	Transaminases increased^b^ (Grade 3)	D36	D36: 309 U/L	D36: 193 U/L D37: 247 U/L	D36: 9 µmol/L	**FU (D120):** ALT: 29 U/L; AST: 27 U/L	Yes	Recovered/resolved
4 (Arm 1a)	ALT increased (Grade 4)	D42	D42: 1109 U/L	D42: 665 U/L	D42: 32 µmol/L	**FU (D61):** ALT: 120 U/L; AST: 99 U/L; BIL: 19 µmol/L	Yes	Recovered/resolved
5 (Arm 1a)	ALT increased (Grade 3)	D88	D88: 462 U/L D157: 266 U/L	D88: 197 U/L D157: 225 U/L	D88: 15 µmol/L D157: 24 µmol/L	**FU (D183):** ALT: 98 U/L; AST: 102 U/L	Yes	Recovered/resolved
	ALT increased (Grade 3)	D157					Yes	Recovered/resolved
	AST increased (Grade 3)	D157					Yes	Recovered/resolved
6 (Arm 1a)	ALT increased (Grade 3)	D70	D70: 404 U/L	D70: 310 U/L	D70: 12 µmol/L	**EOCT (D84):** ALT: 168 U/L; AST: 137 U/L	Yes	Not recovered/resolved
	AST increased (Grade 3)	D70					Yes	Not recovered/resolved
7 (Arm 1a)	ALT increased^b^ (Grade 3)	D57	D57: 87 U/L D71: 566 U/L	D57: 59 U/L D71: 301 U/L	D57: 7 µmol/L D71: 9 µmol/L	**FU (D99):** ALT: 424 U/L; AST: 132 U/L	Yes	Not recovered/resolved
	AST increased^b^ (Grade 3)	D57					Yes	Recovered/resolved
8 (Arm 1a)	ALT increased (Grade 2)	D44	D44: 80 U/L	D44: 56 U/L	D44: 9 µmol/L	**FU (D310):** ALT: 17 U/L; AST: 30 U/L	Yes	Recovered/resolved
	ALT increased^b^ (Grade 3)	D142	D142: 201 U/L	D142: 109 U/L	D142: 12 µmol/L		Yes	Recovered/resolved
9 (Arm 2)	ALT increased^b^ (Grade 4)	D41	D41: 753 U/L	D41: 585 U/L	D41: 14 µmol/L	**FU (D118):** ALT: 82 U/L; AST: 100 U/L	Yes	Recovered/resolved
	AST increased^b^ (Grade 3)	D41					Yes	Recovered/resolved
10 (Arm 2)	ALT increased (Grade 3)	D71	D71: 275 U/L	D71: 330 U/L	D71: 12 µmol/L	**EOCT (D83):** ALT: 186 U/L; AST: 53 U/L	Yes	Not recovered/resolved
	AST increased (Grade 3)	D71					Yes	Not recovered/resolved
11 (Arm 2)	AST increased (Grade 2)	D57	Plasma: D57: 32 U/L D73: 102 U/L D85: 141 U/L D101: 257 U/L D108: 402 U/L	Plasma: D57: 41 U/L D73: 67 U/L D85: 78 U/L D101: 128 U/L D108: 183 U/L	Plasma: D57: 10 µmol/L D73: 9 µmol/L D85: 12 µmol/L D101: 12 µmol/L D108: 10 µmol/L	**FU (D124):** ALT: 68 U/L; AST: 31 U/L	Yes	Recovered/resolved
	ALT increased (Grade 3)	D73					Yes	Recovered/resolved

*AE* adverse event, *ALT* alanine aminotransferase, *AST* aspartate aminotransferase, *BIL* bilirubin, *CTCAE* Common Terminology Criteria for Adverse Events, *D* day, *DILI* drug-induced liver injury, *EOCT* end of combination treatment, *FU* follow-up, *Max* maximum.

^a^Possibly causally related to any study treatment, as assessed by the investigator.

^b^Listed as a serious adverse event.

### Pharmacokinetics and pharmacodynamics in the dose-expansion phase

The pharmacokinetics of each compound were similar to those previously reported in gefitinib and durvalumab monotherapy trials,^37,38^ indicating no drug–drug interaction between gefitinib and durvalumab (Supplementary Table 4 and Supplementary Fig. 2). No treatment-emergent anti-drug antibodies were observed for durvalumab when combined with gefitinib. Complete inhibition of soluble PD-L1, a pharmacodynamic biomarker for durvalumab activity, was observed in all patients (Supplementary Fig. 3), consistent with durvalumab monotherapy at this dose.^30^

### Efficacy in the dose-expansion phase

ORR was 63.3% and 70.0% in Arms 1 + 1a and Arm 2, respectively (Table 2). DCR was 100.0% in Arms 1 + 1a and 90.0% in Arm 2, indicating that almost all patients in the dose-expansion phase achieved disease control.

**Table 2:** Antitumour activity (tumour response analysis set).

Dose-expansion phase	Arms 1 + 1a (*N* = 30)	Arm 2 (*N* = 10)
BOR, *n* (%)		
CR	0	0
PR	19 (63.3)	7 (70.0)
SD ≥8 weeks	10 (33.3)	2 (20.0)
Unconfirmed response	1 (3.3)	0
PD	0	1 (10.0)
ORR, % (95% CI)	63.3 (43.9–80.1)	70.0 (34.8–93.3)
DCR, % (95% CI)	100.0 (88.4–100.0)	90.0 (55.5–99.7)
DCR at 16 weeks, % (95% CI)	90.0 (73.5–97.9)	80.0 (44.4–97.5)
Median DoR, months (95% CI)	9.2 (3.7–14.0)	12.6 (5.5–20.4)
Median PFS, months (95% CI)	10.1 (5.5–15.2)	12.0 (2.7–15.6)

*BOR* best overall response, *CI* confidence interval, *CR* complete response, *DCR* disease control rate, *DoR* duration of response, *N* number of patients assigned to treatment, *ORR* overall response rate, *PD* progressive disease, *PFS* progression-free survival, *PR* partial response *SD* stable disease.

**Fig. 3:**
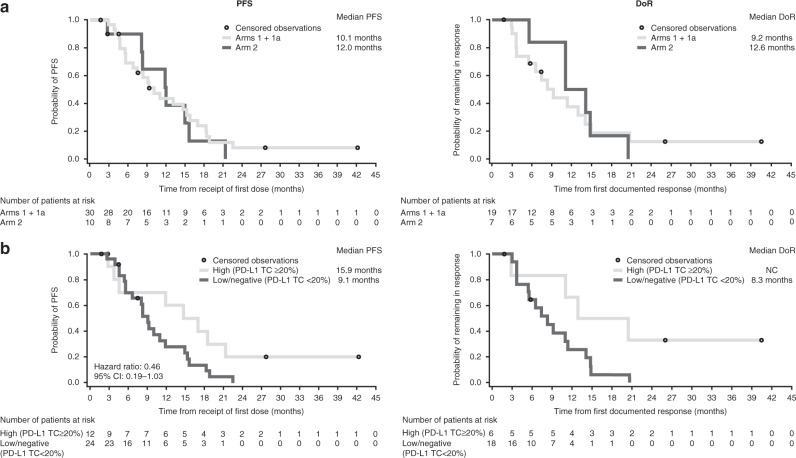
Progression-free survival and duration of response. **a** Overall and **b** by PD-L1 expression (tumour response analysis set). PD-L1 TC expression (high: ≥20%; low/negative: <20%) was determined at baseline. CI confidence interval, DoR duration of response, *NC* not calculable, PD-L1 programmed cell death ligand-1, PFS progression-free survival, TC tumour cell.

**Fig. 4:**
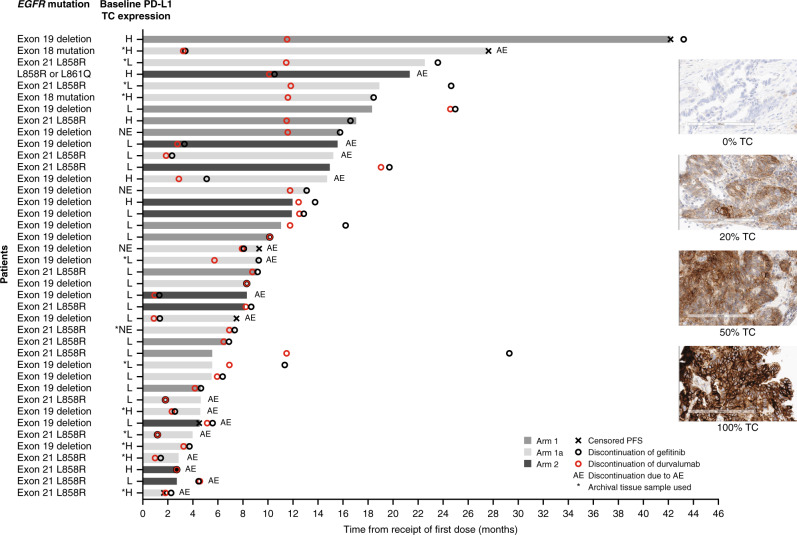
PFS for individual patients in the dose-expansion phase (tumour response analysis set). Baseline PD-L1 TC ≥20% was indicative of high PD-L1 expression. AE adverse event, EGFR epidermal growth factor receptor, H high (PD-L1 TC ≥20%), L low/negative (PD-L1 TC <20%), NE not evaluable (no sample or <100 TCs), PD-L1 programmed cell death ligand-1, PFS progression-free survival, TC tumour cell.

Median duration of response was 9.2 months (95% confidence interval [CI]: 3.7–14.0) in Arms 1 + 1a and 12.6 months (95% CI 5.5–20.4) in Arm 2, while median PFS was 10.1 (95% CI: 5.5–15.2) in Arms 1 + 1a and 12.0 months (95% CI: 2.7–15.6) in Arm 2 (Fig. 3). However, given the small number of patients in Arm 2, these results should be interpreted with caution. The duration of PFS for individual patients in Arms 1 + 1a and Arm 2 is shown in Fig. 4. In an exploratory analysis, a trend towards favourable PFS was noted in patients expressing baseline PD-L1 TC ≥20% (*N* = 12 vs. 24; hazard ratio: 0.46; 95% CI: 0.19–1.03); Figs. 3 and 4). It was not possible to compare the median duration of response for patients expressing PD-L1 TC ≥20%, due to small patient numbers.

Of note, in a post hoc analysis, patient baseline data were assessed for evidence of central nervous system (CNS) metastases, which were reported as present in 14/40 patients at baseline. At least 23.1% of patients who had not reported CNS metastases at baseline had progression to new CNS lesions. Furthermore, for patients with baseline CNS metastases, 50% progressed due to CNS lesions. Unfortunately, few patients consented to long-term survival follow-up, yielding insufficient events at the time of data cut-off to provide an interpretable Kaplan–Meier overall survival estimate.

## Discussion

In this trial combining gefitinib with durvalumab immunotherapy, no synergistic efficacy signal was detected, and the incidence of AEs was higher than expected. The incidence of hepatic AEs with concurrent gefitinib plus durvalumab was notably higher than previously reported for gefitinib (2.4%) and durvalumab (2.8%) monotherapy.^5,30,39^ The observed transaminitis led to treatment discontinuation in >25% of patients, potentially compromising the dose intensity of the EGFR inhibitor. Although some patients were successfully managed with dose interruption or corticosteroids, this remained a significant concern during the course of the trial. Owing to the small numbers of patients in the study, it is difficult to draw definite conclusions around the impact of AEs on PFS, which was no better than historical reports of PFS with gefitinib monotherapy (Supplementary Fig. 4). It is possible that PFS was reduced due to toxicity; however, when considering the width of the CIs, the PFS in each of the groups was not dissimilar despite the observed differences in the number of patients discontinuing treatment due to AEs. This hepatic phenomenon suggests a potential synergy in liver toxicity. Interestingly, treatment-related AEs associated with elevations in ALT and AST have been observed in other first-generation EGFR TKI/immunotherapy combinations, such as erlotinib plus atezolizumab (14.3%),^40^ erlotinib plus nivolumab (14.3%)^11^ and erlotinib plus pembrolizumab (25.0%),^41^ and particularly gefitinib plus pembrolizumab (71.4%).^41^ Recruitment to the latter trial was stopped after seven patients enrolled, due to frequency and severity of transaminitis. Hepatotoxicity may be due to the formation of reactive gefitinib metabolites in the liver,^42^ leading to inflammation when combined with an immune checkpoint inhibitor.

Since this study was initiated, the third-generation EGFR TKI osimertinib is now available for first-line treatment of patients with *EGFR*-mutant metastatic NSCLC.^1,43^ In contrast to gefitinib, osimertinib had a high incidence of interstitial lung disease when combined with durvalumab (13/34; 38%).^44^ This resulted in early termination of the subsequent Phase 3 CAURAL combination trial.^45^ In the first 13 ALK+ patients treated with nivolumab plus crizotinib, 5 developed severe hepatic toxicities leading to drug discontinuation.^46^ Of these, two patients died and the presence of severe hepatic toxicities may have contributed to death. Taken together, the safety profiles associated with EGFR/ALK TKI plus PD-(L)1 inhibitor combinations have generally shown somewhat higher toxicity than expected, reflecting the potential exacerbation of intrinsic but typically minimal toxicities of various TKIs.

In this Phase 1 trial, there was no improvement in PFS or ORR compared to that previously reported with gefitinib monotherapy in similar patient populations.^5,39^ Similar trials of TKI plus PD-1 axis inhibitors have also had no clear evidence of therapeutic synergy, compared with EGFR TKI monotherapy.^40,41^ In a small Phase 1b study of erlotinib plus atezolizumab, response rate of 75% and median PFS of 15 months was observed; likewise erlotinib plus either pembrolizumab or nivolumab had modestly favourable median PFS and ORR.^11^ Perhaps due to this unfavourable efficacy-to-toxicity ratio, the clinical investigation of EGFR TKI plus PD-1 axis inhibitor combinations has largely curtailed in the past 2 years, particularly for TKI-naive patients. To the best of our knowledge, no Phase 3 trials with an EGFR TKI plus PD-1 inhibitor for EGFR TKI-naive patients are currently planned or actively accruing.

Although our trial did observe numerically greater improvement in median PFS in patients with baseline PD-L1 TC ≥20%, this finding needs to be interpreted with caution due to the small sample size. Similarly, an association between PD-L1 expression and improved efficacy was suggested with another EGFR TKI/immunotherapy combination in KEYNOTE-021, in which partial response was reported in all patients with baseline PD-L1 tumour proportion scores ≥50%.^41^ Although PD-L1 TC ≥25% was associated with efficacy of durvalumab monotherapy in patients with *EGFR-*mutant NSCLC with acquired TKI resistance,^30^ a recent Phase 2 study found that pembrolizumab monotherapy was ineffective for the treatment of EGFR TKI-naive patients with PD-L1 TC ≥1%, in which many were ≥50%.^47^ Although higher PD-L1 and tumour mutational burden may also be predictive for early relapse for *EGFR*-mutant patients while receiving EGFR TKI monotherapy, these reports remain largely exploratory and inconclusive.^24,27,48^

In summary, results from this Phase 1 study do not support the combination of TKIs and anti-PD-L1 in the EGFR TKI treatment-naive setting. Given the diverse array of resistance mutations and clonal heterogeneity for EGFR TKI-resistant patients, it is possible that the relapsed/refractory setting may be a more opportune setting for T cell or immune checkpoint-based therapy. Further trials are warranted to elucidate the role of anti-PD-1/PD-L1 agents in the treatment paradigm for patients with *EGFR*-mutant NSCLC and determine whether baseline tumour PD-L1 expression is predictive of improved durability of response.

## Supplementary information

Supplementary Information

## Data Availability

Data underlying the findings described in this manuscript may be obtained in accordance with AstraZeneca’s data sharing policy described at https://astrazenecagrouptrials.pharmacm.com/ST/Submission/Disclosure.
