# Supplementary material for: A Phase 1 study of gefitinib combined with durvalumab in EGFR TKI-naive patients with EGFR mutation-positive locally advanced/metastatic non-small-cell lung cancer
Source: Br J Cancer. 2020 Oct 5;124(2):383–90. doi: 10.1038/s41416-020-01099-7 (PMC7852511; doi:10.1038/s41416-020-01099-7)
Supplement: Supplementary file 1 — Supplementary Information [file 41416_2020_1099_MOESM1_ESM.docx]

**Supplementary Information**

## Supplemental Data 1

### Patients

All patients had at least one lesion that could be accurately assessed at baseline by computed tomography or magnetic resonance imaging, and was suitable for repeated assessments. In the dose-expansion phase, target lesions were not previously irradiated, were measurable at baseline (≥20 mm in the longest diameter, or in the shortest diameter for lymph node lesions) and were suitable for accurate repeated measurements and serial biopsy. Tumour responses were determined using Response Evaluation Criteria in Solid Tumours v1.1. The clinical trial protocol was conducted in accordance with GCP and ICH guidelines.

Patients receiving chemotherapy, immunotherapy, biological or hormonal therapy for the treatment of cancer, either concurrently or within 28 days of the first dose of study medication, were not eligible. However, patients that received local treatment of isolated legions for palliative intent (e.g., by local surgery or radiotherapy), but not wide- or limited-field radiotherapy (within 4 weeks and 2 weeks of the first dose of study medication, respectively) were eligible. Additional exclusion criteria included: inadequate bone barrow reserve or organ function; a history of (or clinically active) interstitial lung disease or pneumonitis which required steroid treatment; pre-existing idiopathic pulmonary fibrosis evidenced by computed tomography scan at baseline; active or prior documented autoimmune or inflammatory disease within the past 3 years; and the use of medications or herbal medications known to be moderate or strong inducers of cytochrome P450 3A4.

### Study assessments

Safety and tolerability were assessed using: the type, incidence, and severity of adverse events (AEs; coded using the Medical Dictionary for Regulatory Activities [MedDRA] version 21.0, and graded by the National Cancer Institute Common Terminology Criteria for Adverse Events [CTCAE] v4.03); laboratory data, including clinical chemistry, hematology, coagulation, thyroid function, urinalysis, and creatinine clearance assessments, and any associated abnormalities (including dose-limiting toxicities [DLTs]); vital signs; and changes in electrocardiogram and echocardiogram.

Blood samples were collected to determine the concentrations of gefitinib and durvalumab in plasma and serum, respectively, during concomitant treatment periods. A non-compartmental analysis was used to calculate durvalumab pharmacokinetic parameters using Phoenix WinNonlin. The pharmacokinetics of durvalumab were assessed after a single dose and after multiple doses in combination with gefitinib. The steady-state exposure of gefitinib was assessed after multiple doses in combination with durvalumab. Immunogenicity was determined by evaluating the number and percentage of patients who developed detectable anti-drug antibodies, and was used to determine the impact of anti-drug antibodies on overall durvalumab pharmacokinetics, determined from the concentration of durvalumab. Durvalumab pharmacodynamics were assessed by measuring the inhibition of soluble programmed cell death ligand-1 (PD-L1) expression levels in the serum.

At each visit, patient responses were determined as being a complete response (CR), partial response (PR), stable disease (SD), progressive disease (PD) or not evaluable depending on disease status versus baseline and previous assessments. Objective response rate (ORR) was defined as the percentage of patients with measurable disease at baseline with ≥1 documented CR or PR prior to disease progression. Duration of response (DoR) was calculated from the last date of the first documented CR or PR until the first incidence of disease progression or death. Disease control rate (DCR) was defined as the percentage of patients with ≥1 documented CR, PR or SD ≥8 weeks prior to disease progression, and DCR at 16 weeks was defined as the percentage of patients who either achieved CR or PR 16 weeks after commencing combination therapy or demonstrated SD for at least 16 weeks after commencing combination therapy. Duration of progression-free survival (PFS) was calculated from the first administration of study treatment to the first documentation of PD or death from any cause. Overall survival (OS) was calculated from receipt of the first dose until the date of death from any cause (1).

### Statistical analyses

For safety variables, data from all cycles of initial treatment were combined in the presentation of data. All safety variables were presented individually for each patient. AEs were categorised by MedDRA v21.0 system organ class, preferred term, and CTCAE v4.03 grade, and were also summarised by dose group/expansion arm.

Individual durvalumab serum concentrations and pharmacokinetics parameters were summarised by cohort using descriptive statistics. For inclusion in the pharmacokinetics analysis set, patients were required to have received at least one dose of study medication and have at least one measurable pharmacokinetics concentration, supported by the dates and times of study medication administration in the 2 days prior to sample provision, and of sample provision itself. Additionally, for concentration data from pharmacokinetic samples to be included in the pharmacokinetics analysis set, dosing data from 2 days prior to sample collection was required. Pharmacokinetics and durvalumab pharmacodynamics samples were collected in parallel, and pharmacodynamics were assessed using the pharmacodynamics analysis set (all patients who received at least one dose of durvalumab and had at least one soluble PD-L1 (sPD-L1) assessment). Immunogenicity was assessed using the anti-drug antibodies analysis (ADA) set (all patients who received at least one dose of durvalumab and had at least one ADA assessment).

Tumour response data (including ORR, DoR, DCR, and PFS) were summarised by dose-escalation or dose-expansion phase arm. OS analysis was based on the safety population; results were due to be presented using Kaplan-Meier plots and summary statistics, including median OS.

### REFERENCES

1. Fairman D, Narwal R, Liang M, Robbins P, Schneider A, Chavez C*, et al*. Pharmacokinetics of MEDI4736, a fully human anti-PDL1 monoclonal antibody, in patients with advanced solid tumors. *J Clin Oncol* 2014;**32**: (abstract).

## Supplemental Data 2


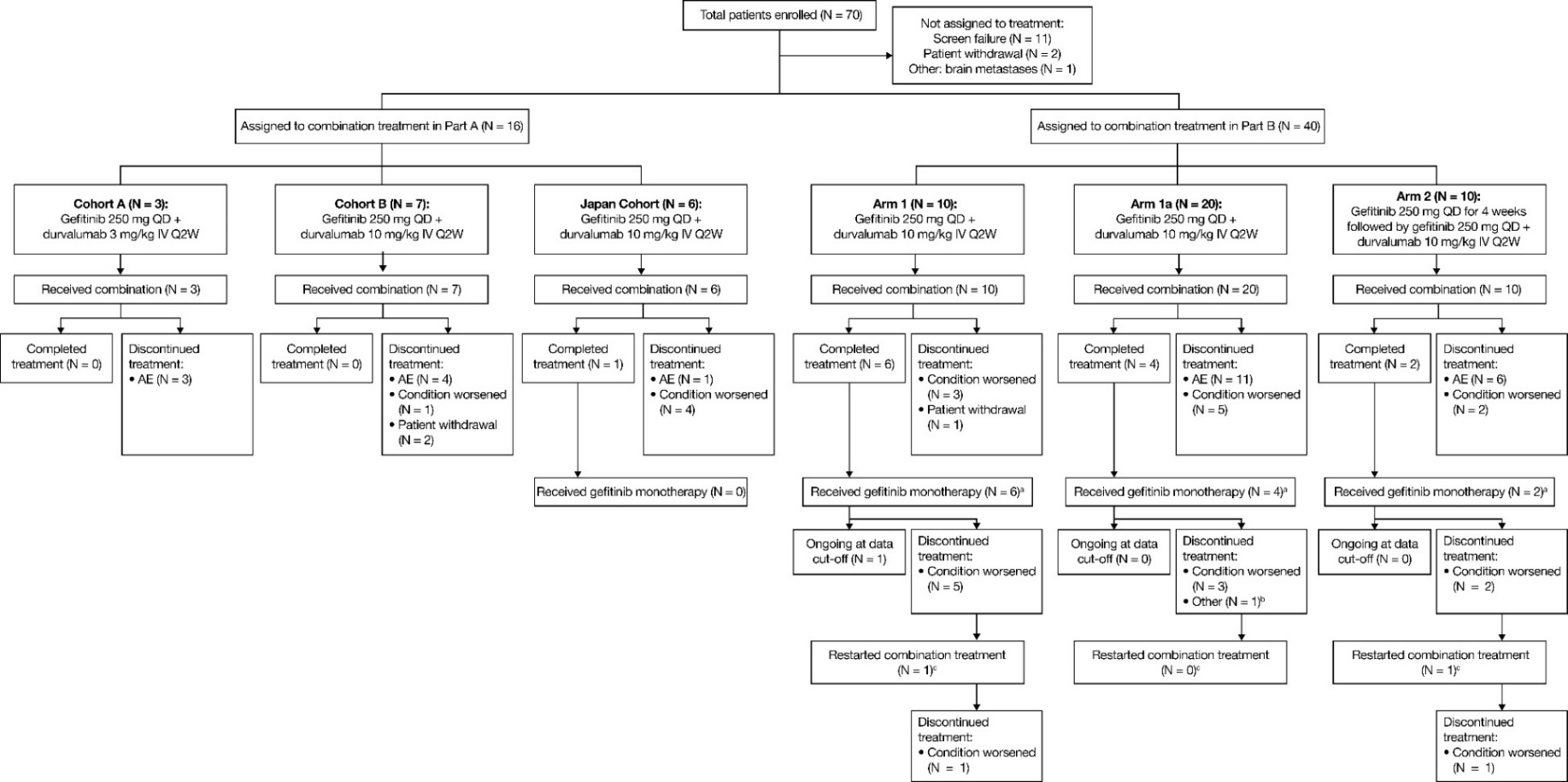
**Fig. 1** Patient disposition.

^a^After the initial 12-month combination treatment period. ^b^Patient switched to insurance-provided gefitinib. ^c^In the post-combination treatment period.
AE, adverse event; IV, intravenous; N, number of patients; QD, once daily; Q2W, once every 2 weeks.

## Supplemental Data 3

### Table 1. Patient demographics and baseline characteristics (safety population).

|  | Dose-escalation Phase | | | Dose-expansion Phase | | | Dose-expansion |
| --- | --- | --- | --- | --- | --- | --- | --- |
|  | Cohort A  (n = 3) | Cohort B (n = 7) | Japan Cohort (n = 6) | Arm 1  (n = 10) | Arm 1a (n = 20) | Arm 2 (n = 10) | Total (n = 40) |
| Median age, years  (min, max) | 63.0  (55, 67) | 57.0  (41, 83) | 66.5  (46, 78) | 54.5  (27, 68) | 60.5  (45, 78) | 66.0  (57, 76) | 61.5  (27,78) |
| Sex, n (%) |  |  |  |  |  |  |  |
| - Male | 0 (0.0) | 4 (57.1) | 1 (16.7) | 5 (50.0) | 10 (50.0) | 5 (50.0) | 20 (50.0) |
| - Female | 3 (100.0) | 3 (42.9) | 5 (83.3) | 5 (50.0) | 10 (50.0) | 5 (50.0) | 20 (50.0) |
| Race, n (%) |  |  |  |  |  |  |  |
| - Asian | 1 (33.3) | 3 (42.9) | 6 (100.0) | 5 (50.0) | 11 (55.0) | 5 (50.0) | 21 (52.2) |
| - Black or African American | 0 (0.0) | 0 (0.0) | 0 (0.0) | 0 (0.0) | 2 (10.0) | 0 (0.0) | 2 (5.0) |
| - White | 2 (66.7) | 4 (57.1) | 0 (0.0) | 5 (50.0) | 7 (35.0) | 5 (50.0) | 17 (42.5) |
| ECOG PS, n (%) |  |  |  |  |  |  |  |
| - 0 | 1 (33.3) | 4 (57.1) | 0 (0.0) | 3 (30.0) | 5 (25.0) | 2 (20.0) | 10 (25.0) |
| - 1 | 2 (66.7) | 3 (42.9) | 6 (100.0) | 7 (70.0) | 15 (75.0) | 8 (80.0) | 30 (75.0) |
| *EGFR* mutation, n (%) |  |  |  |  |  |  |  |
| - Exon 18 mutation | 0 (0.0) | 0 (0.0) | 0 (0.0) | 0 (0.0) | 2 (10.0) | 0 (0.0) | 2 (5.0) |
| - Exon 19 deletion | 1 (33.3) | 3 (42.9) | 0 (0.0) | 6 (60.0) | 9 (45.0) | 5 (50.0) | 20 (50.0) |
| - Exon 21 L858R | 0 (0.0) | 1 (14.3) | 4 (66.7) | 4 (40.0) | 8 (40.0) | 4 (40.0) | 16 (40.0) |
| - L858R or L861Q | 0 (0.0) | 0 (0.0) | 0 (0.0) | 0 (0.0) | 0 (0.0) | 1 (10.0) | 1 (2.5) |
| Prior treatments, n (%) |  |  |  |  |  |  |  |
| - Immunotherapy | 0 (0.0) | 0 (0.0) | 1 (16.7) | 0 (0.0) | 0 (0.0) | 0 (0.0) | 0 (0.0) |
| - Cytotoxic chemotherapy | 0 (0.0) | 2 (28.6) | 6 (100.0) | 0 (0.0) | 1 (5.0) | 0 (0.0) | 1 (2.5) |
| - Systemic therapy | 2 (66.7) | 3 (42.9) | 6 (100.0) | 0 (0.0) | 1 (5.0) | 2 (20.0) | 3 (7.5) |
| - Radiotherapy | 1 (33.3) | 1 (14.3) | 4 (66.7) | 1 (10.0) | 5 (25.0) | 0 (0.0) | 6 (15.0) |
| - Other | 0 (0.0) | 1 (14.3) | 0 (0.0) | 0 (0.0) | 0 (0.0) | 0 (0.0) | 0 (0.0) |

ECOG PS, Eastern Cooperative Oncology Group performance status; EGFR, epidermal growth factor receptor; max, maximum; min, minimum; n, number of patients.

## Supplemental Data 4

### Table 2. Summary of adverse events (safety population).

|  | Dose-escalation phase | | Dose-expansion phase | | |
| --- | --- | --- | --- | --- | --- |
|  | Cohort A  (N = 3) | Cohort B + Japan Cohort  (N = 13) | Arm 1  (N = 10) | Arm 1a  (N = 20) | Arm 2  (N = 10) |
| Patients, n (%) | 3 (100.0) | 13 (100.0) | 10 (100.0) | 20 (100.0) | 10 (100.0) |
| AE (all-causality) | 3 (100.0) | 13 (100.0) | 10 (100.0) | 20 (100.0) | 10 (100.0) |
| Grade ≥3 AE (all-causality) | 3 (100.0) | 8 (61.5) | 5 (50.0) | 15 (75.0) | 8 (80.0) |
| AE with outcome death (all-causality) | 1 (33.3) | 1 (7.7) | 0 (0.0) | 1 (5.0)^a^ | 0 (0.0) |
| SAE (all-causality) | 3 (100.0) | 5 (38.5) | 3 (30.0) | 13 (65.0) | 5 (50.0) |
| AE (all-causality) leading to discontinuation^b^ | 3 (100.0) | 5 (38.5) | 0 (0.0) | 11 (55.0) | 6 (60.0) |
| AE (treatment-related^c^) | 3 (100.0) | 12 (92.3) | 10 (100.0) | 20 (100.0) | 10 (100.0) |
| Grade ≥3 AE (treatment-related^c^) | 2 (66.7) | 5 (38.5) | 4 (40.0) | 11 (55.0) | 7 (70.0) |
| AE with outcome death (treatment-related^c^) | 0 (0.0) | 0 (0.0) | 0 (0.0) | 0 (0.0) | 0 (0.0) |
| SAE (treatment-related^c^) | 1 (33.3) | 2 (15.4) | 1 (10.0) | 7 (35.0) | 4 (40.0) |
| AE (treatment-related^c^) leading to discontinuation^b^ | 2 (66.7) | 3 (23.1) | 0 (0.0) | 10 (50.0) | 6 (60.0) |

## ^a^Death related to disease under investigation; ^b^Discontinuation of gefitinib and/or durvalumab; ^c^Possibly causally related to any study treatment, as assessed by the investigator. AE: adverse event; n: number of patients; SAE: serious adverse event.Supplemental Data 5

### Table 3. Summary of Causally-Related Adverse Events (Safety Population)

|  | Dose-escalation phase^a^ | | Dose-expansion phase^b^ | | |
| --- | --- | --- | --- | --- | --- |
|  | Cohort A | Cohort B + Japan Cohort | Arm 1 | Arm 1a | Arm 2 |
| **Patients with any caually-related AE, n (%)^c^** | 3 (100.0) | 12 (92.3) | 10 (100.0) | 20 (100.0) | 10 (100.0) |
| Diarrhoea | 3 (100.0) | 5 (38.5) | 9 (90.0) | 12 (60.0) | 6 (60.0) |
| ALT increased | 2 (66.7) | 4 (30.8) | 7 (70.0) | 10 (50.0) | 60 (60.0) |
| AST increased | 2 (66.7) | 4 (30.8) | 4 (40.0) | 8 (40.0) | 5 (50.0) |
| Dry skin | 1 (33.3) | 4 (30.8) | 3 (30.0) | 5 (25.0) | 5 (50.0) |
| Fatigue | 2 (66.7) | 3 (23.1) | 3 (30.0) | 1 (5.0) | 1 (10.0) |
| Nausea | 1 (33.3) | 4 (30.8) | 6 (60.0) | 2 (10.0) | 1 (10.0) |
| Vomiting | 0 (0.0) | 4 (30.8) | - | - | - |
| Amylase increased | 2 (66.7) | 2 (15.4) | 1 (10.0) | 2 (10.0) | 1 (10.0) |
| Dermititis acneiform | 0 (0.0) | 4 (30.8) | 2 (20.0) | 6 (30.0) | 2 (20.0) |
| Decreased appetitie | 1 (33.3) | 2 (15.4) | 3 (30.0) | 3 (15.0) | 1 (10.0) |
| Myalgia | 0 (0.0) | 3 (23.1) | - | - | - |
| Pruritus | 0 (0.0) | 3 (23.1) | 5 (50.0) | 7 (35.0) | 6 (60.0) |
| Pyrexia | 1 (33.3) | 2 (15.4) | - | - | - |
| Rash maculo-papular | 1 (33.3) | 2 (15.4) | 1 (10.0) | 2 (10.0) | 2 (20.0) |
| Rash | - | - | 6 (60.0) | 9 (45.0) | 5 (50.0) |
| Stomatitis | - | - | 2 (20.0) | 2 (10.0) | 3 (30.0) |
| Paronychia | - | - | 3 (30.0) | 1 (5.0) | 1 (10.0) |
| Palmar-plantar erythrodysaesthesia syndrome | - | - | 1 (10.0) | 1(5.0) | 2 (20.0) |

^a^Occuring in at least 3 subjects overall; ^b^Occuring in at least 10% of subjects overall; ^c^Causally related to any study treatment, as assessed by the Investigator.

AE: adverse event; ALT: alanine aminotransferase; AST: aspartate aminotransferase.

## Supplemental Data 6

### Table 4. Gefitinib plasma concentration in the dose-expansion phase (pharmacokinetics analysis set).

|  |  | Time point (pre-infusion^a^) | | | | | |
| --- | --- | --- | --- | --- | --- | --- | --- |
| Group | Statistic | Cycle 1 Day 15 | Cycle 4 Day 1 | Cycle 6 Day 1 | Cycle 8 Day 1 | Cycle 10 Day 1 | Cycle 12 Day 1 |
| Arm 1  (N = 10) | n^b^ | 9 | 10 | 8 | 6 | 8 | 6 |
|  | Geometric mean (ng/mL) | 158.33 | 188.49 | 199.34 | 189.31 | 217.38 | 229.77 |
|  | %CV | 36.1 | 46.4 | 47.0 | 48.2 | 50.1 | 87.6 |
| Arm 1a  (N = 20) | n^b^ | 19 | 2 | 10 | 3 | 6 | 1 |
|  | Geometric mean (ng/mL) | 227.34 | 273.95 | 204.91 | 146.16 | 195.14 | 91.90 |
|  | %CV | 63.1 | 19.5 | 87.7 | 15.5 | 65.8 | NC |
| Arm 2  (N = 10) | n^b^ | 7 | 6 | 5 | 5 | 4 | 3 |
|  | Geometric mean (ng/mL) | 317.93 | 275.63 | 250.08 | 251.56 | 267.25 | 284.00 |
|  | %CV | 41.1 | 29.8 | 36.6 | 18.7 | 23.7 | 21.2 |

^a^Pre-infusion samples required during the study treatment period were to be obtained within 60 minutes prior to dosing; ^b^Number of patients was identical to the number of quantifiable values (greater than the lower limit of quantification) at all listed time points.
CV: coefficient of variation; N: number of patients assigned to treatment; n: number of patients in analysis; NC: not calculable.

## Supplemental Data 7

### Fig. 2 Durvalumab serum concentration in the dose-expansion phase (pharmacokinetics analysis set).


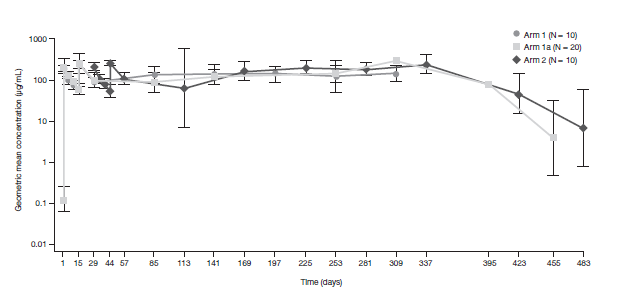


Data are geometric mean serum concentration ± standard deviation on the log scale.
N: number of patients assigned to treatment.

## Supplemental Data 8

### Fig. 3 Soluble PD-L1 serum concentrations (pharmacodynamics analysis set).


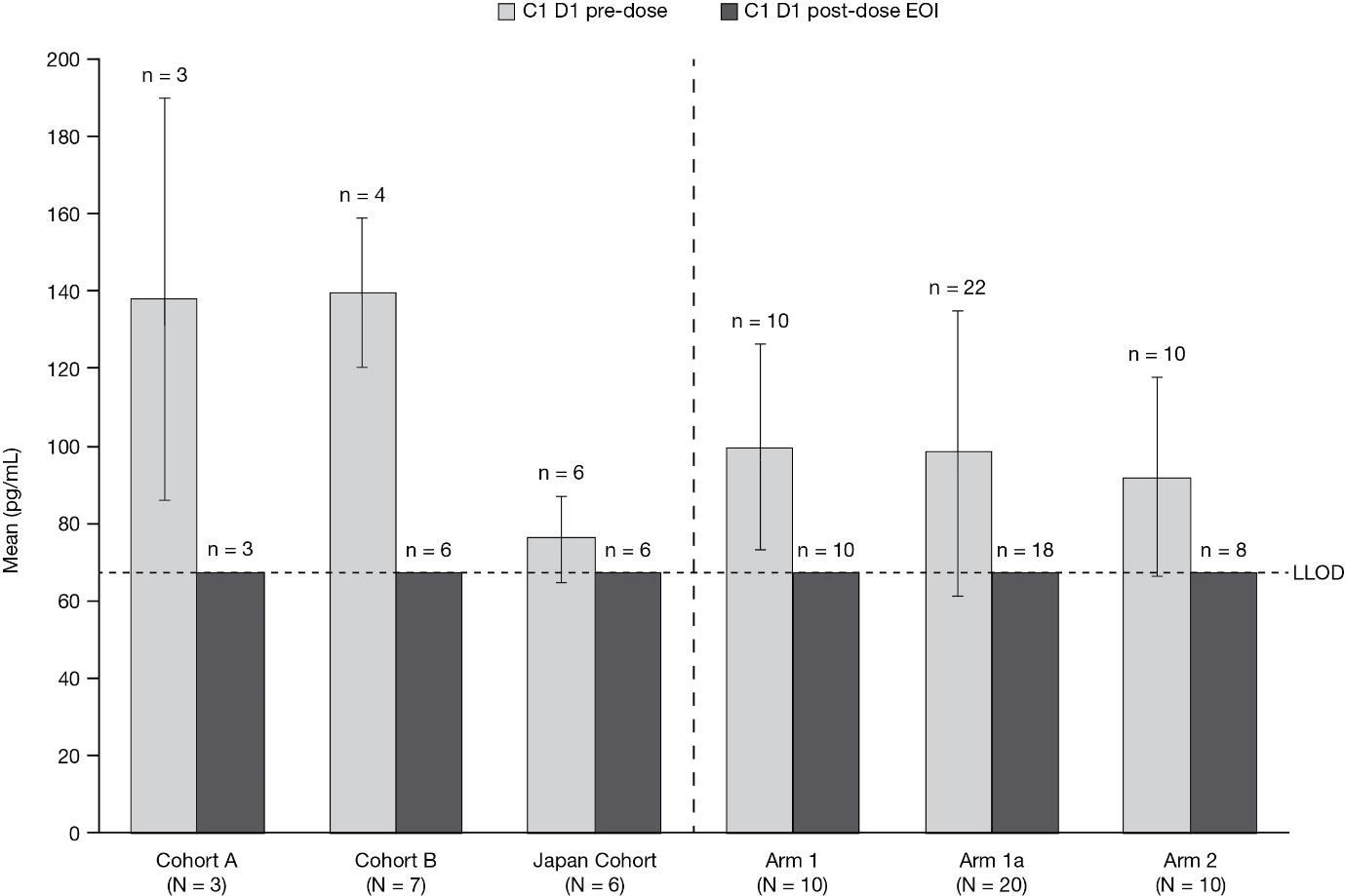

Data are mean serum concentration ± standard deviation, presented for all patients who received at least one dose of durvalumab and had at least one soluble PD-L1 assessment (by patient group). Data from C1D1 are shown here. Patients showed sPD-L1 detection at C1D1 pre-dose, but these levels fell below LLOD at C1D1 post-dose.
End of infusion: EOI; LLOD: lower limit of detection; N: number of patients assigned to treatment; n: number of patients with data; PD-L1, programmed cell death ligand-1.

## Supplemental Data 9

### Fig. 4 Cross-study comparison of median PFS with gefitinib plus durvalumab vs. gefitinib monotherapy.


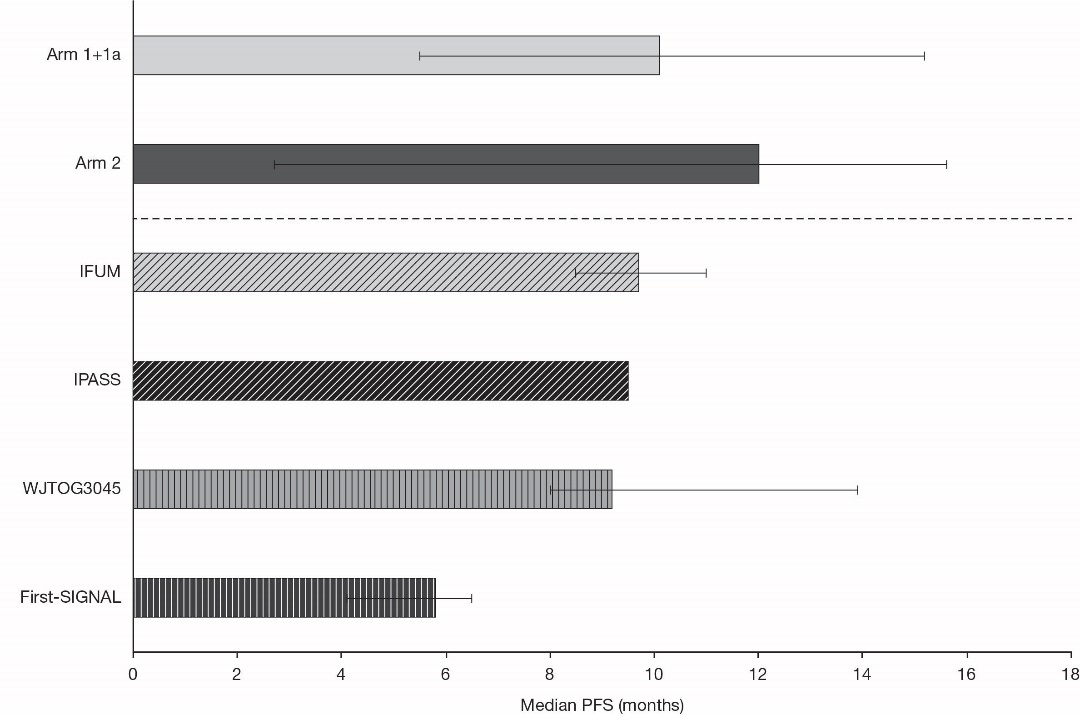


Data are median PFS ± 95% CIs. These results should be interpreted with caution due to differences in study design and enrolled patient populations.
CI: confidence interval; PFS: progression-free survival.
